# An Account of a Peculiarity of Vision in a Girl at East Dereham in Norfolk

**Published:** 1787

**Authors:** J. S. Webster

**Affiliations:** Surgeon at East Dereham


### V. *An Account of a Peculiarity of Viſion in a Girl at Eaſt Dereham in Norfolk. Communicated in a Letter to Dr.* Simmons *by Mr.* J. S. Webſter, *Surgeon at Eaſt Dereham.*

I BEG leave, Sir, to communicate to you the following account of a remarkable defect of ſight, which (with your approbation) I wiſh to ſee inſerted in the London Medical Journal. — I think it right, however, firſt to obſerve to you, that my appointment of ſurgeon to the Houſe of Induſtry, in which the girl, who is the ſubject of the caſe, is at preſent maintained, has afforded me frequent opportunities of examining into the particulars attending her defect of ſight; and as in all my inquiries I have been upon my guard againſt deception, ſo likewiſe I doubt not but you will give me credit when I aſſure you I am as unwilling to deceive as to be deceived.

Helen Bunnett, or, as ſhe is commonly called, the *owl-eyed girl*, is thirteen years old, of a fair complexion, with brown hair, and has all her life enjoyed a good ſtate of health. She was born in a workhouſe belonging to Eaſt Dereham, in the county of Norfolk; but is now ſupported in a Houſe of Induſtry belonging to the hundreds of Milford and Launditch, in the fame county.

This girl has from her infancy laboured under a peculiarity of vifion. What particularly ftrikes one's attention, on her entering a room in the day time, is, her looking towards the ground, and her eyes appearing, as it were, funk in her head; fo much fo, that the whole ball of the eye feems loft within its orbit, and of courfe the eyelid fo covers it, that you would at firft imagine the humours of the eye had efcaped from their coats.

No appearance of difeafe is perceptible in the coats of the eye. The choroid is of a whitifh or light gray colour. The iris is peculiarly perfect. The pupils are entirely black; and the appearance of each eye is the fame.

I firft put her faculty of vifion to the teft by exhibiting large objects before her eyes, fuch as a watch, a broad button, the key of a door, &c. Thefe fhe certainly was able to diftinguifh, though with difficulty; and I obferved that fhe is very near fighted.

I next offered to her bottles filled with medicines of different colours, fuch as blue vitriolic water, vegeto-mineral water, and others; but in attempting to diftinguifh thefe fhe, in general, ral, failed. I then preſented to her view ſmall objects, ſuch as a ſixpence, a ſhilling, pins, &c.; but theſe ſhe could not diſcover at all.

Upon cloſing the windows, and darkening the room ſuddenly, I had my attention fixed upon her eyes, which inſtantly dilated, and the pupils became as perfect, and as large in proportion, as in any human body whatever; on the contrary, upon opening the windows as ſuddenly as I before had cloſed them, the pupils became inſtantly contracted, and the balls of the eyes appeared, as it were, ſunk. I then cloſed her eyelids, and rubbed them frequently, but without obſerving any appearance of dilatation in the eyes. Having now again darkened the room ſo much that I could not myſelf diſtinguiſh objects, I had in readineſs the ſame bottles of medicines as before, and likewiſe ſome pieces of cloth of different colours that I had offered to her when the windows were not cloſed, and which ſhe had then not been able to diſtinguiſh: but upon my again offering the ſame to her in the darkened room, I was agreeably ſurpriſed to find that ſhe could tell me the colours of the different fluids in the bottles, as well as the quantities therein contained, and alſo the various colours of the cloths, cloths, excepting of thofe which we may term mixed cloths; and perhaps in thefe fhe failed not from a want of perception, but from not being fufficiently practifed in the diftinctions of complicated colours. I likewife took a pin, and having dropped it upon the ground, at a confiderable diftance from that part of the room where fhe ftood, changed places with her, and defired her to look for the pin, which fhe very foon found. All the time the room remained darkened her eyes were fully dilated, and continued equally fo, neither contracting nor increafing in their dilatation.

The expreffion of owl-eyed girl, which I have made ufe of, is not a term given to her by me, but is a diftinction fhe goes by among the paupers in general in the houfe where fhe now is.

I lately afked her the following queftions, which I fhall give you, with her anfwers, as I minuted them upon the fpot:

Q. "How is your eyefight when in the "fun?

A. "I cannot then fee in the leaft.

Q. "Are your eyes ever painful to you?

A. "They are very painful in fummer and "hot weather.

Q. "In what direction do you look when "you wish to distinguish any thing?

A. "From the corners of my eyes, as one "cross-eyed."

She has informed me likewise, that she can distinguish objects as well by moonlight, or in the twilight, as in the dark.

*East Dereham,*

August 12, 1787

